# Supplementary material for: Characterization of the Kenyan Honey Bee (Apis mellifera) Gut Microbiota: A First Look at Tropical and Sub-Saharan African Bee Associated Microbiomes
Source: Microorganisms. 2020 Nov 3;8(11):1721. doi: 10.3390/microorganisms8111721 (PMC7692941; doi:10.3390/microorganisms8111721)
Supplement: Supplementary file 1 [file microorganisms-08-01721-s001.zip › microorganisms-974258.SUPFigures.revised.docx]

**Supplementary figures**

**
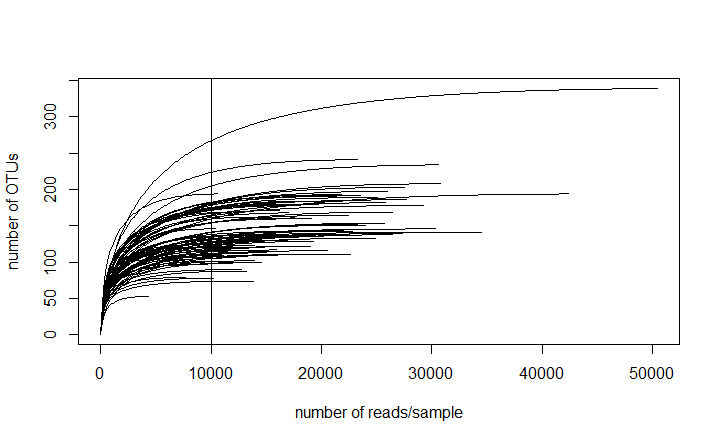
**

**Figure S1: Rarefaction curve (OTUs per reads/samples).** The rarefaction value is 10,000 reads.

**
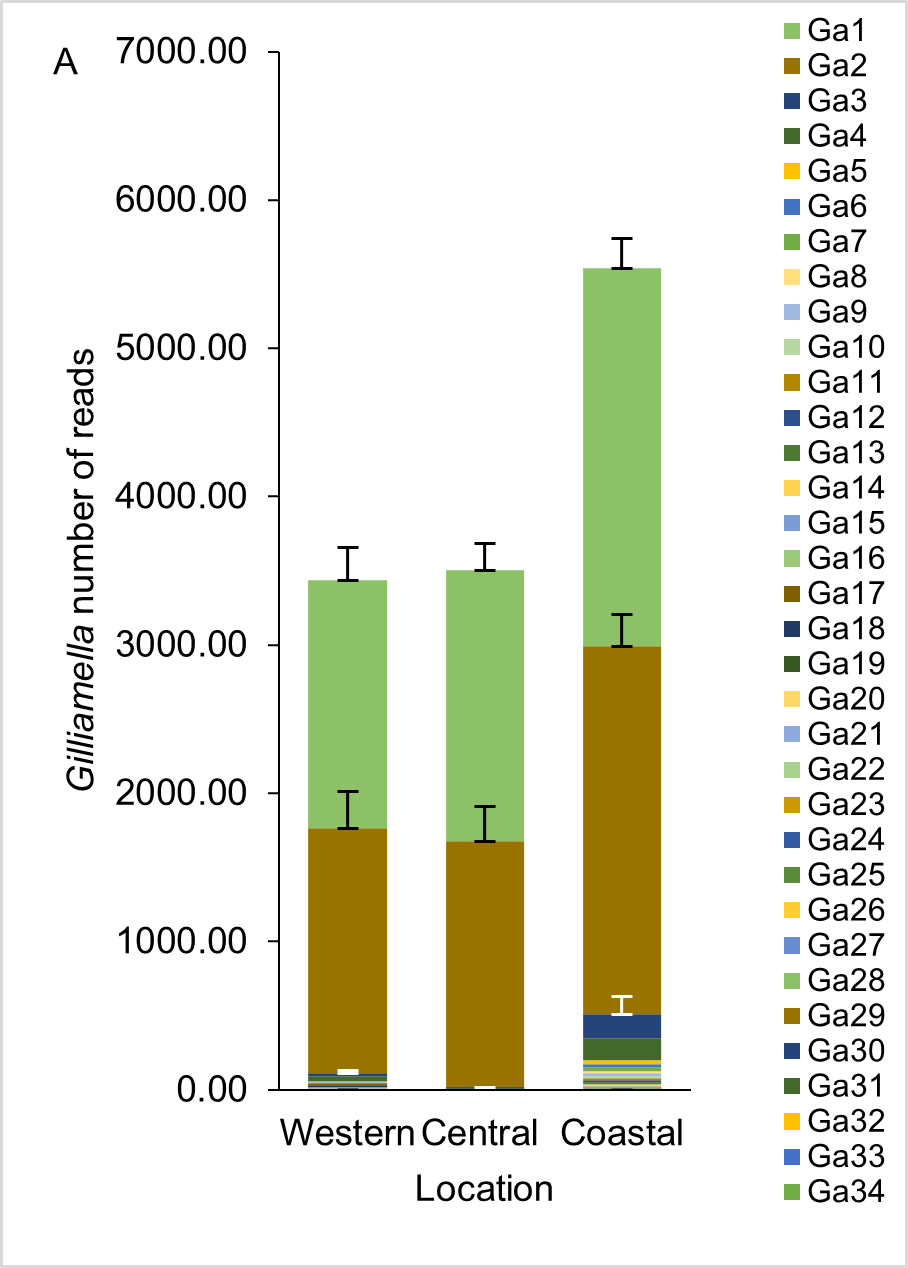

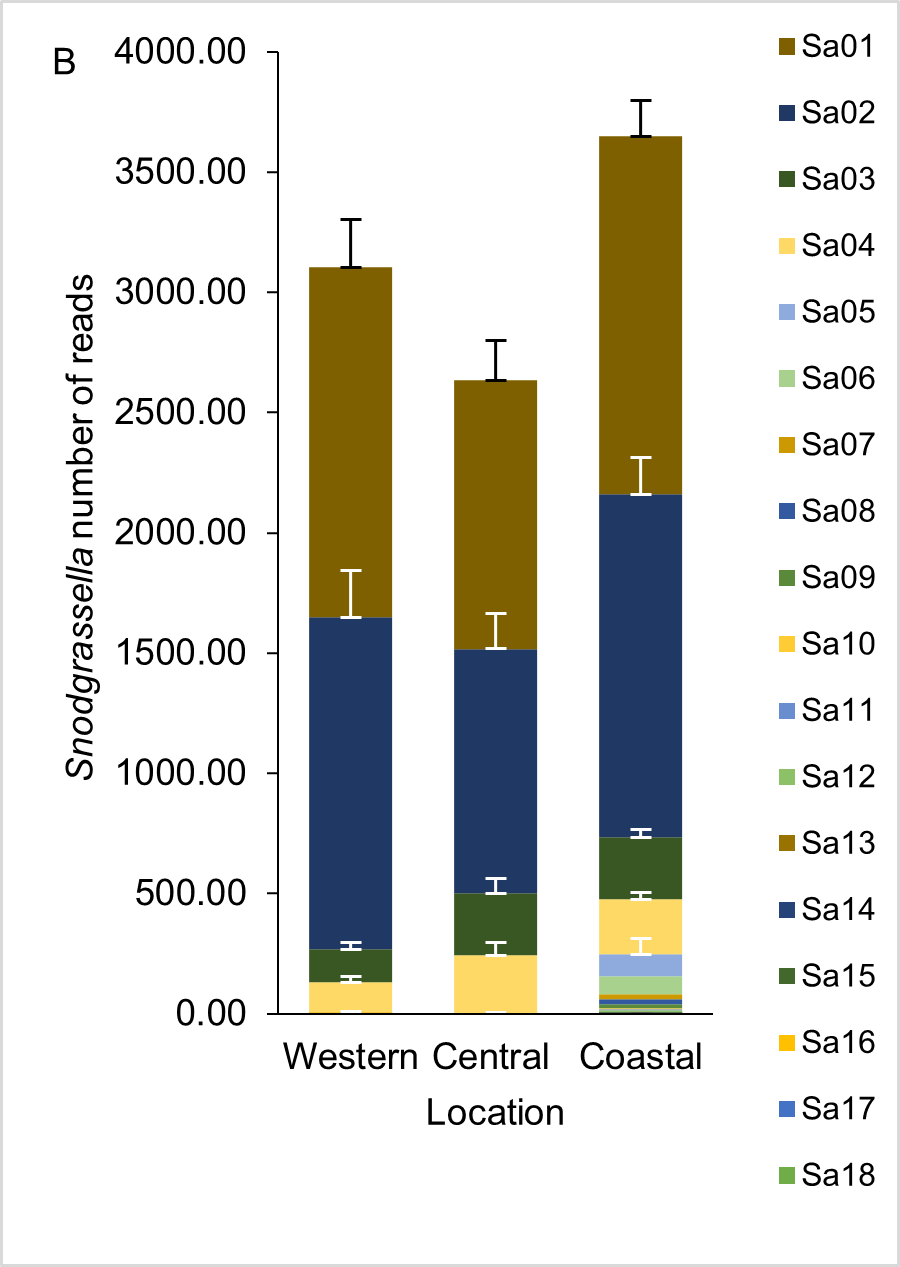
**

**
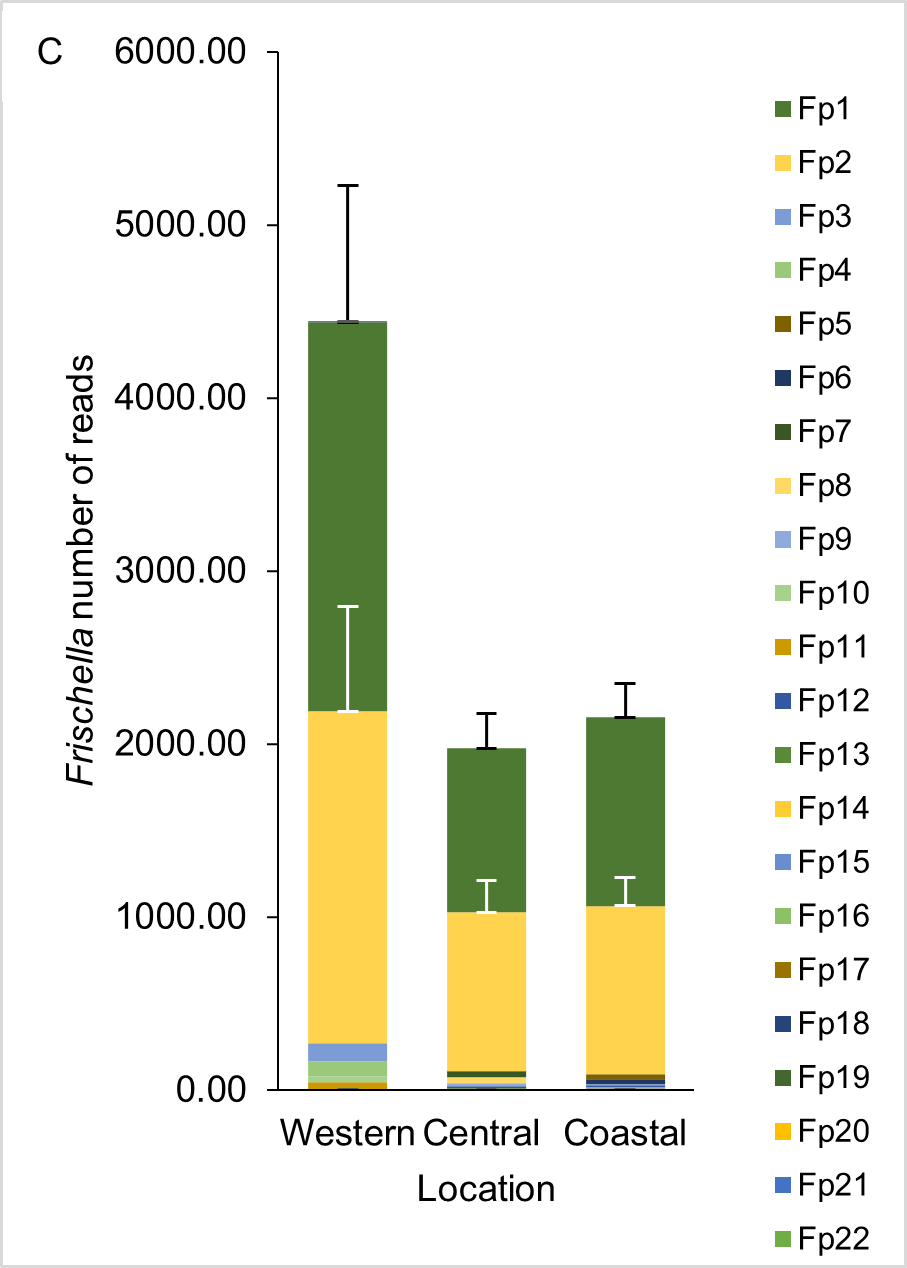

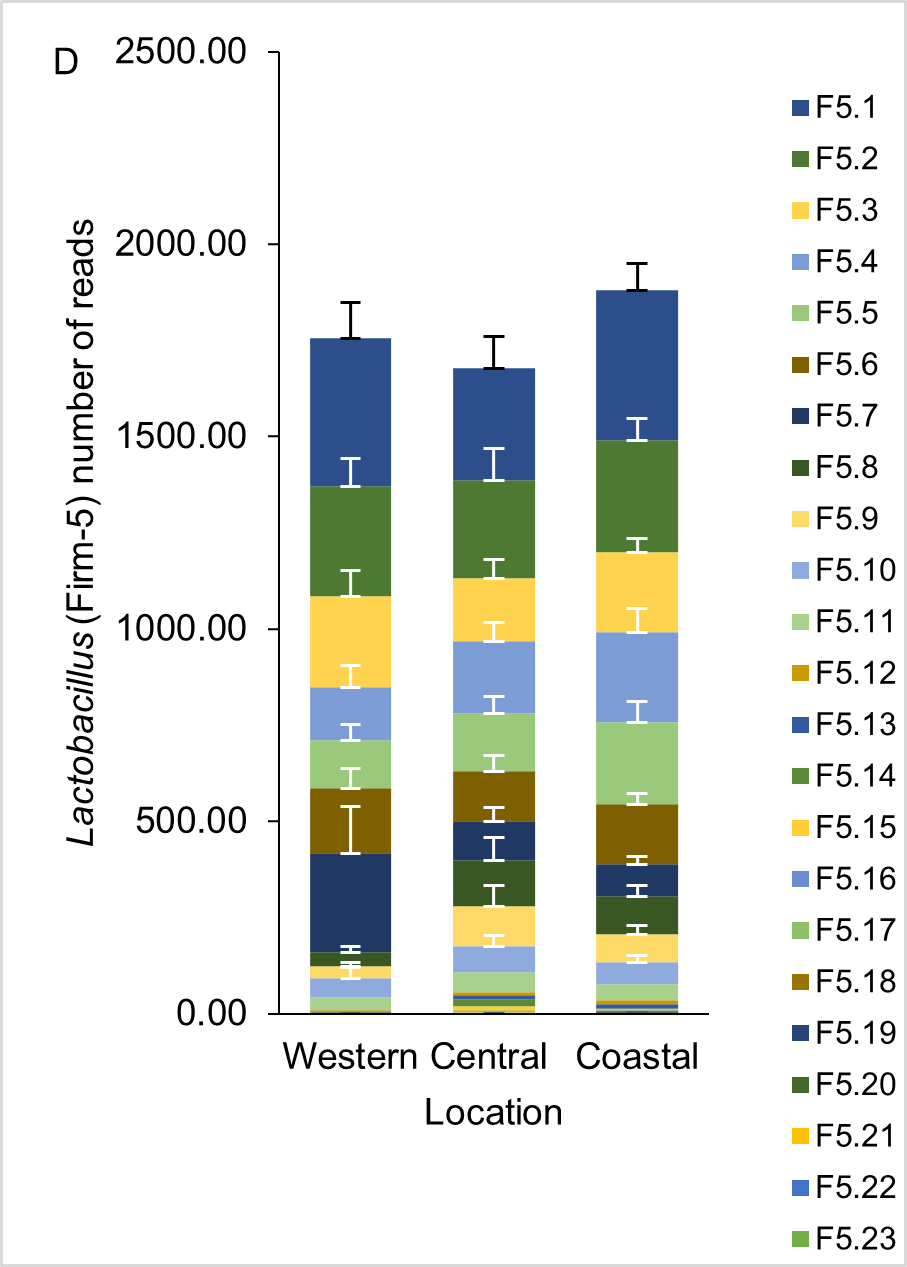
**

**
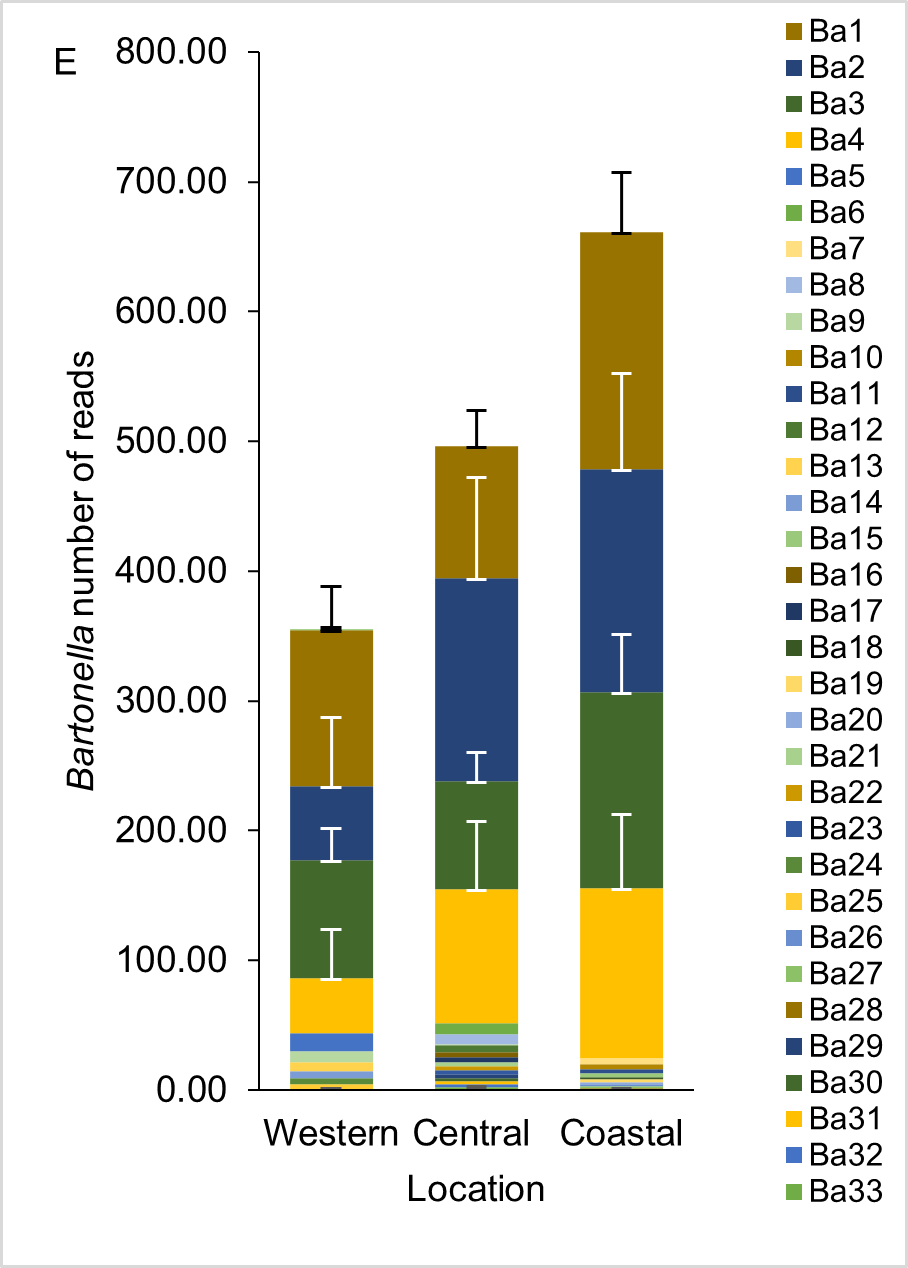

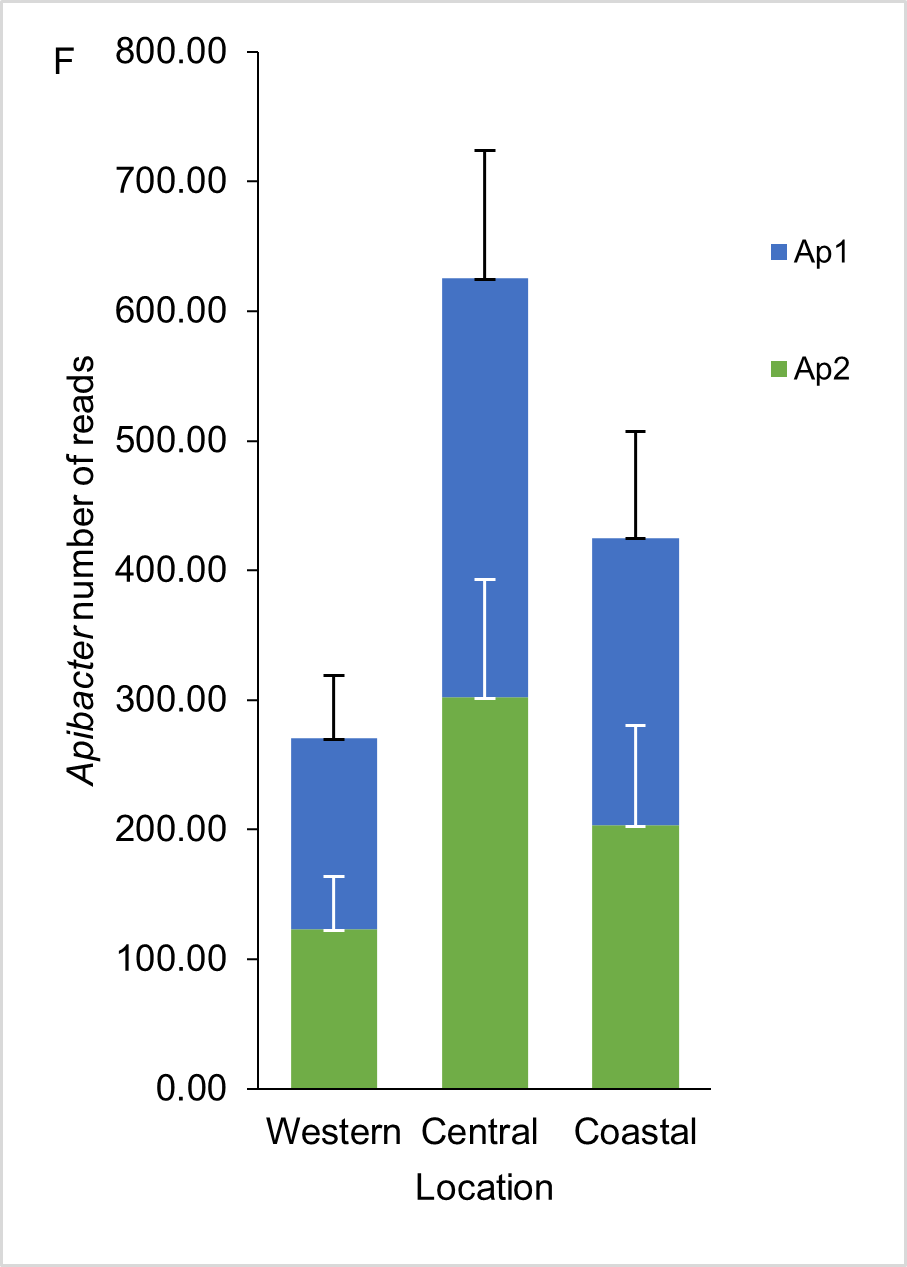
**

**
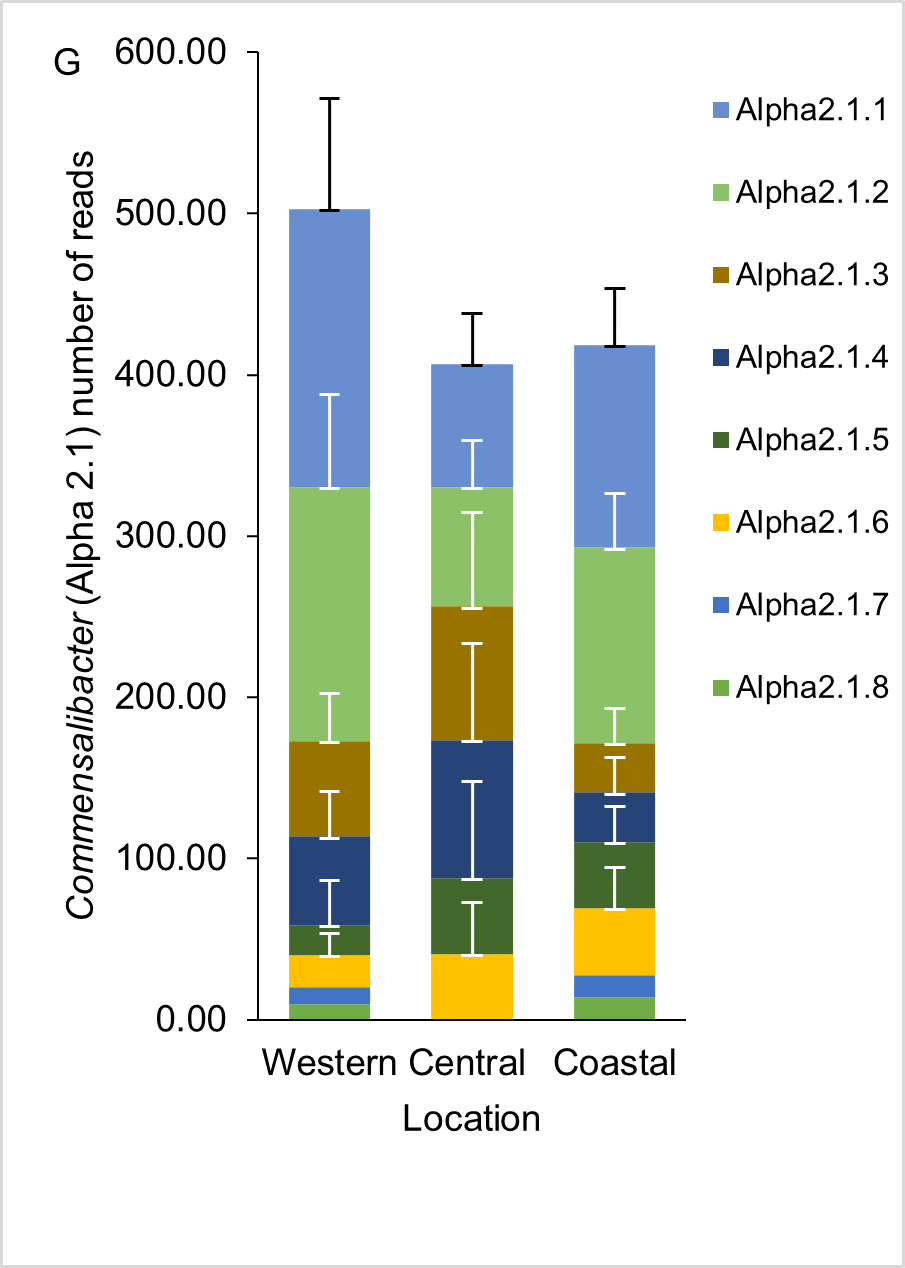

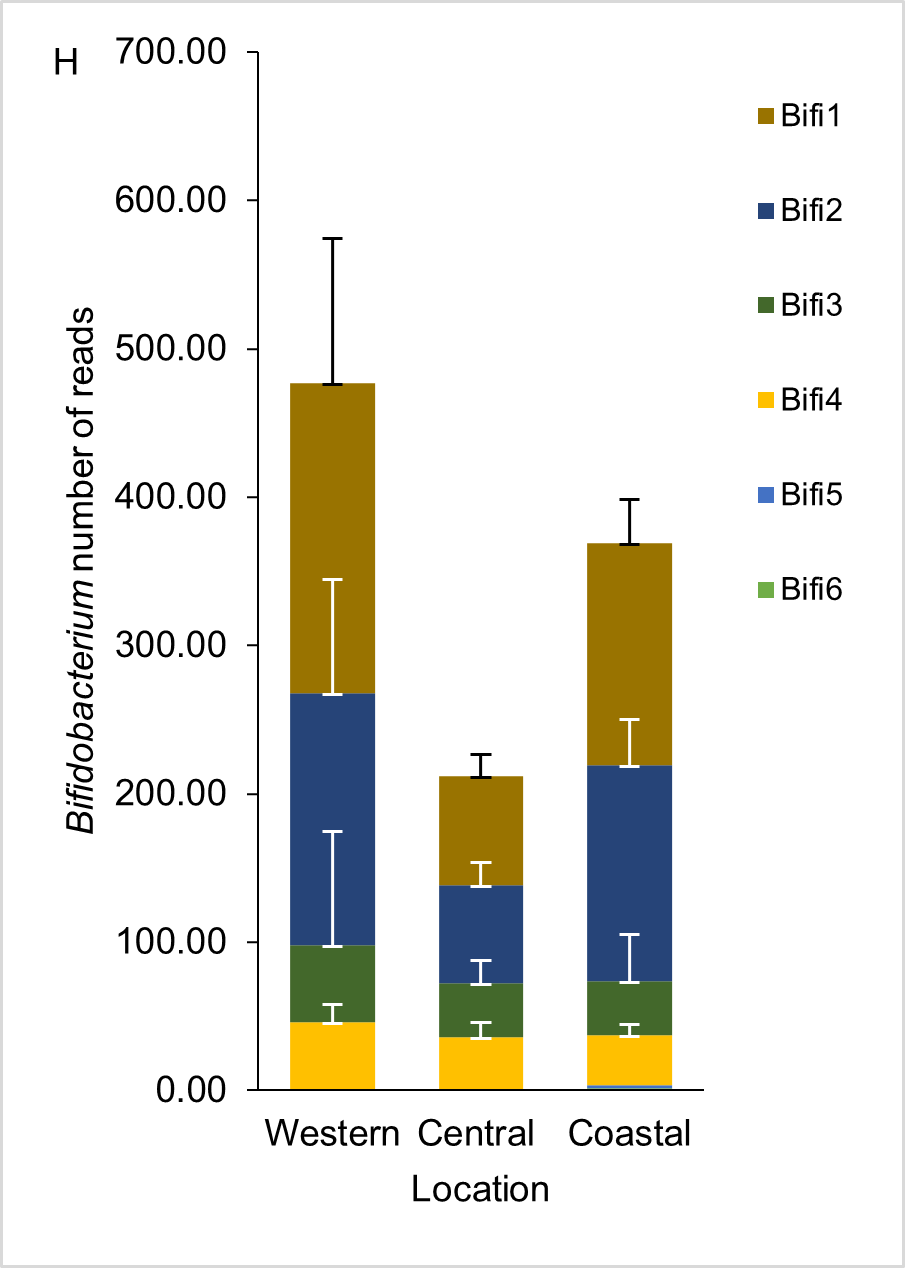
**

**
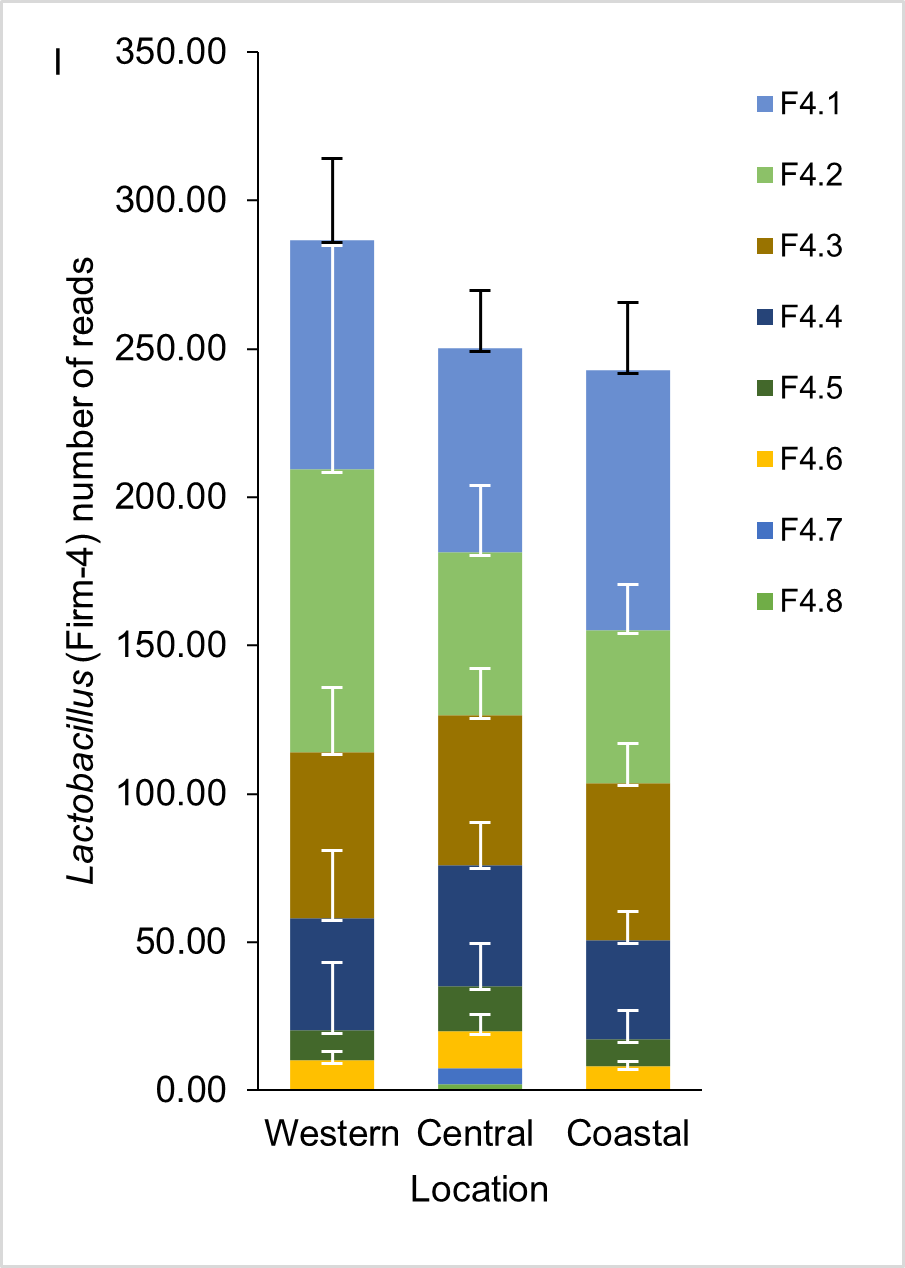

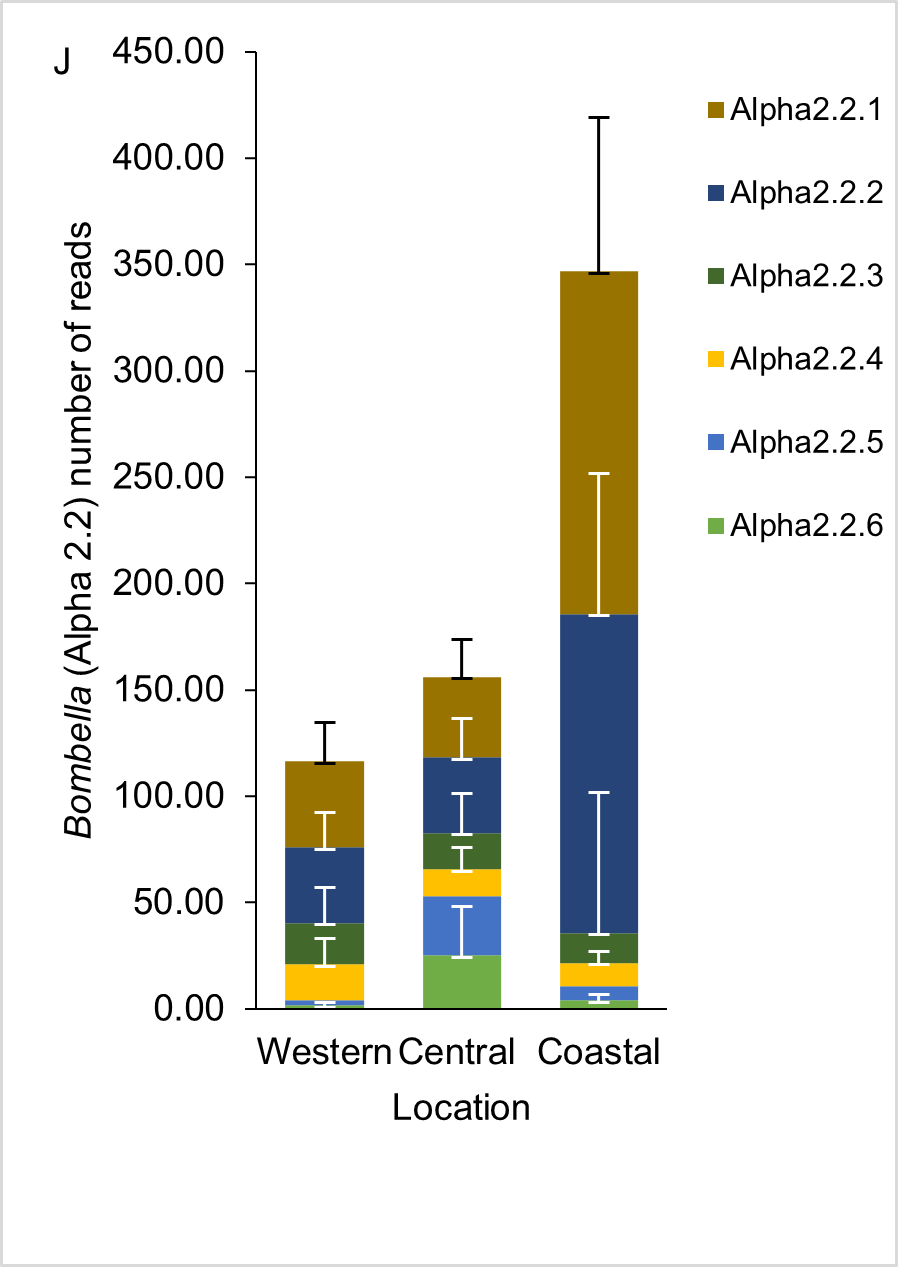
**

**Figure S2: Amplicon sequence variant (ASV) relative abundance of the main bacteria core members**.

**Table S2: Most abundant environmental bacterial abundance and prevalence.**

| **Environmental bacterial abundance (%) and prevalence (%)** | | | | | | | |
| --- | --- | --- | --- | --- | --- | --- | --- |
|  |  | **Western** | | **Central** | | **Coastal** | |
|  |  | **Abundance** | **Prevalence** | **Abundance** | **Prevalence** | **Abundance** | **Prevalence** |
| Environmental bacterial genus | *Pseudomonas* | 3.1 | 87.2 | 1.2 | 77.9 | 2.3 | 62.8 |
|  | *Acidovorax* | 3.4 | 87.2 | 2.4 | 81.4 | 0.6 | 61.6 |
|  | *Spiroplasma* | 2.2 | 83.7 | 3.9 | 76.7 | 0.0 | 54.7 |
|  | *Gluconobacter* | 1.9 | 93.0 | 1.5 | 90.7 | 1.8 | 89.5 |
|  | *Acinetobacter* | 0.0 | 79.1 | 3.8 | 75.6 | 0.0 | 55.8 |
|  | *Klebsiella* | 1.2 | 86.0 | 0.6 | 79.1 | 0.7 | 64.0 |
|  | *Fructobacillus* | 1.4 | 88.4 | 0.8 | 84.9 | 0.3 | 70.9 |
|  | *Enterobacter* | 0.8 | 84.9 | 0.3 | 74.4 | 0.4 | 58.1 |
|  | *Weissella* | 1.8 | 82.6 | 0.1 | 73.3 | 0.0 | 52.3 |
|  | *Tatumella* | 0.6 | 90.7 | 0.5 | 87.2 | 0.3 | 77.9 |

**Table S3: PERMANOVA analysis showed no bacterial community variation among locations.** R^2^ = 0.03074, P = 0.198.

| **Group 1** | **Group 2** | **F.Model** | **R^2^** | **p-value** | **p-adjusted** |
| --- | --- | --- | --- | --- | --- |
| Western | Coastal | 1.378317 | 0.02282801 | 0.136 | 0.408 |
| Western | Central | 1.110891 | 0.02638013 | 0.346 | 1.000 |
| Coastal | Central | 1.570429 | 0.02324137 | 0.060 | 0.180 |

**Table S4: Pairwise comparisons of bacterial relative abundance showed significant variation of *Gilliamella* among locations.** Statistical significance are indicated with different letters.

| **Pairwise Comparison** | | | | | | |
| --- | --- | --- | --- | --- | --- | --- |
|  |  | **F-value** | **P-value** | **Western** | **Central** | **Coastal** |
| Bacterial genus | *Gilliamella* | 3.105 | 0.0501 | b | ab | a |
|  | *Snodgrassella* | 0.18 | 0.836 | a | a | a |
|  | *Frischella* | 2.156 | 0.122 | a | a | a |
|  | *Lactobacillus* (Firm-5) | 0.675 | 0.512 | a | a | a |
|  | *Bartonella* | 0.38 | 0.685 | a | a | a |
|  | *Apibacter* | 1.756 | 0.179 | a | a | a |
|  | *Commensalibacter* (Alpha 2.1) | 0.908 | 0.407 | a | a | a |
|  | *Bifidobacter* | 1.821 | 0.168 | a | a | a |
|  | *Lactobacillus* (Firm-4) | 0.776 | 0.464 | a | a | a |
|  | *Bombella* (Alpha 2.2) | 0.461 | 0.632 | a | a | a |
